# Gemcabene, a First-in-Class Hypolipidemic Small Molecule in Clinical Development, Attenuates Osteoarthritis and Pain in Animal Models of Arthritis and Pain

**DOI:** 10.3389/fphar.2018.00471

**Published:** 2018-05-11

**Authors:** Rai A. K. Srivastava, Joseph A. Cornicelli, Bruce Markham, Charles L. Bisgaier

**Affiliations:** ^1^Gemphire Therapeutics Inc., Livonia, MI, United States; ^2^Charles River Laboratories, Inc., Wilmington, MA, United States; ^3^Diapin Therapeutics, Ann Arbor, MI, United States

**Keywords:** gemcabene, inflammation, CRP, IL6, IL-1β, NF-kB, osteoarthritis, hyperalgesia

## Abstract

Our clinical studies have demonstrated that gemcabene, a small molecule in late-stage clinical development, lowers pro-inflammatory acute-phase protein, C-reactive protein (CRP). This observation was further confirmed in a cell-based study showing inhibition of cytokine-induced CRP production. Based on these observations, in the present study, we tested the hypothesis that gemcabene may possess anti-inflammatory activities in animal models of inflammatory disease. Efficacy of gemcabene was investigated in rat models of carrageenan-induced thermal hyperalgesia (CITH), monosodium iodoacetate (MIA)-induced osteoarthritis (OA), and IL-6/IL-6sR-induced inflammation. We also evaluated efficacy of gemcabene in collagen antibody-induced joint swelling and arthritis in BALB/c mice. In CITH rat model, gemcabene administration attenuated paw withdrawal latency (60% at 30 mg/kg/d and 97% at 100 mg/kg/d) and showed improvement in joint swelling (-50% at 30 mg/kg/d) in MIA model of OA. These findings were further corroborated by IL-6/IL-6sR knee injection model in rat, showing 63 and 71% reduction in hind paw weight distribution at 10 and 30 mg/kg/d doses, respectively. In mouse model of monoclonal antibody–induced arthritis, a dose-dependent attenuation of joint swelling was observed. These results demonstrate that the anti-inflammatory activity of gemcabene previously observed in cell-based and in clinical studies also occurred in animal models of inflammation-induced arthritis and hyperalgesia. Thus, in addition to hypolipidemic efficacy, the anti-inflammatory activity of gemcabene may have additional benefits to patients with elevated vascular inflammation.

## Introduction

Gemcabene calcium (also known as gemcabene, CI-1027 and PD 72953-0038) is a small molecule and is the monocalcium salt of a dialkyl ether dicarboxylic acid [6,6′-oxybis (2,2-dimethylhexanoic acid)] and is currently in late-stage clinical development. Gemcabene has shown hypolipidemic properties in preclinical animal models (Bisgaier et al., 1998) as well as in clinical studies(Stein et al., 2016). It inhibits both cholesterol and fatty acid synthesis as determined by the ^14^C-acetate incorporation in hepatocytes (Bisgaier et al., 1998). Clinical studies in humans demonstrated that, in addition to LDL lowering, gemcabene reduces plasma levels of C-reactive protein (CRP) in patients by 53.5% in monotherapy and by 71% in combination with statins (Stein et al., 2016), indicating that this compound may have anti-inflammatory properties. CRP, an acute phase reactant, is produced mainly in the liver in response to inflammation and has been traditionally considered, as a “downstream” indicator of the inflammatory cascade (Arcone et al., 1988). We have reported that gemcabene inhibits cytokine-stimulated CRP production in human hepatoma PLC/PRF/5 (Alexander) cells (Srivastava et al., 2018). Pro-inflammatory cytokines (IL-6 and IL-1β) are known to induce CRP (Li et al., 1990) and associated with inflammatory conditions like pain and OA (Osiri et al., 1998; Brennan and McInnes, 2008; Mori et al., 2011).

Since inflammatory nature of atherosclerosis have been demonstrated both in animal models (Cybulsky and Gimbrone, 1991; Ross, 1999; Cybulsky et al., 2001) as well as in clinical studies (Schonbeck and Libby, 2004; Kinlay et al., 2008; Zhao, 2009) a link has been suggested between high sensitivity C-reactive protein (CRP) with coronary artery disease (CAD) risk (Ridker and Silvertown, 2008; Puri et al., 2013; Halcox et al., 2014; Quaglia et al., 2014). CRP is released during the inflammatory processes (Libby and Ridker, 2004; Ridker et al., 2008), and is recognized as a powerful predictor of cardiovascular risk (Ridker et al., 2000; Lemieux et al., 2001; Saito et al., 2003). This was confirmed by the JUPITER trial, suggesting CRP as a marker of inflammation and atherosclerosis (Libby and Ridker, 2004; Ridker et al., 2008).

In this study, we evaluated anti-inflammatory activities of gemcabene in multiple animal models of inflammatory disease to ascertain anti-inflammatory properties of gemcabene. Carrageenan-induced inflammation in the rat paw is a classical model of edema and hyperalgesia (Guay et al., 2004). Carrageenan injection into the hind paw of rats is commonly used to study inflammation and inflammatory pain. Carrageenan causes edema, an increase in paw volume, and an exacerbated sensitivity to thermal and mechanical stimuli, known as hyperalgesia (Seibert et al., 1994). In the paw edema model, cyclooxygenase-2 (COX-2) levels are elevated (Seibert et al., 1994; Zhang et al., 1997), and COX-2 inhibitors attenuate disease severity (Chan et al., 1995; Zhang et al., 1997). The injection of the metabolic inhibitor MIA (monosodium iodoacetic acid) into joints initiates the pathogenesis of OA, which is characterized by inflammation-induced progressive loss of joint cartilage (van der Kraan et al., 1989; Guingamp et al., 1997; Guzman et al., 2003). OA is a degenerative disorder of the joints that causes pain and functional disability (Henrotin and Kurz, 2007; Krasnokutsky et al., 2008), possibly occurring from joint degeneration resulting from the combination of mechanical stress and biochemical factors (Combe et al., 2004; Marker and Pomonis, 2012). Rheumatoid arthritis (RA) is a systemic disease, characterized by inflammation in multiple joints associated with synovial hyperplasia, as well as concomitant bone and cartilage destruction (Khachigian, 2006; Yasuda, 2006; Nasu et al., 2008). High levels of pro-inflammatory cytokines and proteases that are released from synovial tissue cause changes in chondrocyte metabolism and matrix degradation, which lead to cartilage destruction (Huber et al., 2006; Yasuda, 2006). Interleukin-6 (IL-6) is a pleiotropic cytokine that is involved in the regulation of immune and acute phase responses and is known to contribute to increased pain and hyperalgesia in inflamed tissue (Oka et al., 1995; Arruda et al., 2000). Elevated levels of IL-6 and its soluble receptor (IL-6sR) have been observed in both rheumatoid arthritis (RA) and OA (Kotake et al., 1996; Robak et al., 1998). Injection of IL-6, in combination with its soluble receptor into the knee joint of rats induces a rapid (<1 h) shift in hind paw weight distribution, and can be used as a measure of compound efficacy. CIA is an inducible autoimmune disease that has been used as the gold-standard animal model of RA (Marcelletti et al., 1991). CIA exhibits many of the histological features observed in human RA, including infiltration and proliferation of mononuclear cells, synovial hyperplasia, pannus formation, and destruction of joint cartilage and architecture (Guingamp et al., 1997). However, we chose monoclonal antibody -induced arthritis (MIAA) model over a traditional CIA model because of its ability to induce a CIA-like arthritis in strains of mice other than DBA/1. Since strain-specific marked pharmacological differences have been noticed in mice (Srivastava et al., 1997; Spearow et al., 1999; Spearow et al., 2001) and most knockout mice are made in 129/SvJ, BALB/c or C57BL/6 strains, and not DBA/1, it has been difficult to use knockouts to validate targets for rheumatoid arthritis, primarily because it requires years of backcrossing desired knockout mice onto the DBA/1 background in order to test the effect of a genetic deficiency on the onset or severity of arthritis. In the MAIA model, we found that 129/SvJ, BALB/c, C3H/FeJ, and C57BL/6 strains were similarly responsive as the DBA/1 mice. Since all these animal models represent inflammation-induced progression of disease, gemcabene efficacy to attenuate pathological conditions resulting from inflammation was evaluated in these animal models. Using these animal efficacy models of inflammation, we showed that gemcabene attenuates MIA, MAIA, and IL-6/IL-6sR induced hyperalgesia, OA, and hind paw weight distribution, respectively.

## Experimental Procedures

### Material

Animals were obtained either from Jackson Laboratories or from Charles River Laboratories. Arthrogen-CIA Monoclonal Antibody Blend was procured from Chemicon International, Inc., Temecula, CA, United States). Carrageenan, monoiodoacetate (MIA), hydroxypropyl methylcellulose (HPMC)/, Tween 80 and LPS from *Escherichia coli* Strain 0111B4 were obtained from Sigma Chemical Co., St. Louis, MO, United States. Incapacitance tester was purchased from Linton Instrumentation, United Kingdom. The source of other reagents and equipment are provided in the text where mentioned.

### Animal Housing and Protocol

After arrival from vendors, animals were housed in animal rooms with water and food provided *ad libitum*. The animal study protocols were approved by the Institutional Animal Care and Use Committee (IACUC) of Gemphire Therapeutics Inc. and all guidance was strictly followed to minimize the pain and suffering of animals. Animal dosing were staggered where applicable to measure the endpoints at the same time in order to have meaningful comparison.

### Carrageenan-Induced Thermal Hyperalgesia in the Rat

**FIGURE 1:**
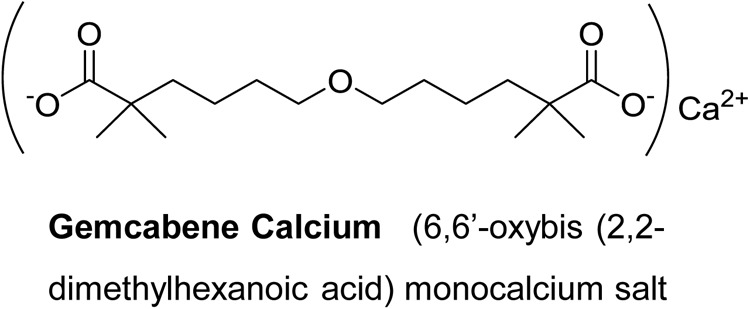
Structure of test substance.

Male Sprague Dawley rats (200–300 g), obtained from Charles River, were housed in groups of 2 under a 12 h light/dark cycle with food and water *ad libitum*. Animals were allowed to acclimate to the test room 1 h prior to testing. To induce inflammation, rats were anesthetized with isoflurane and given an intraplantar injection of carrageenan (2 mg) in saline (0.1 mL). Sham-injected (saline) animals were utilized for determination of baseline paw withdrawal latencies (PWL). Thermal hyperalgesia was assessed using the rat plantar test (apparatus from University of California, San Diego) following a modified method (Hargreaves et al., 1988). PWL were recorded in seconds (“s”). An automatic cut off point of 22.5 s was set to prevent tissue damage. The mean of 2–3 PWL was taken at each time point for both hind paws of each animal. The apparatus was calibrated to give a PWL of approximately 10 s before carrageenan administration. After basal PWL were determined, animals receive an intraplantar 100 μL of 10 mg/mL of lambda carrageenan (Sigma Chemical Co.) into the right hind paw. Lambda carrageenan was dissolved in isotonic saline 5 min prior to injecting. PWL were reassessed following the same protocol 2 h post carrageenan (this time point represented the start of peak hyperalgesia) to ascertain that hyperalgesia had developed. Gemcabene [6-(5-Carboxy-5-methyl-hexyloxy)-2,2-dimethyl-hexanoic acid, calcium salt] (**Figure 1**) prepared in-house was dissolved in 0.5% hydroxypropyl methylcellulose/0.25% Tween-80 and administered orally via oral dosing needles 2.5 h post carrageenan injection, and PWL were taken again at 30- or 60-min intervals post drug administration for 6 h. Indomethacin at 10 mg/ml (formulated in methyl cellulose solution) was used as a reference agent and administered by oral gavage. Hyperalgesia response was measured 2.5 h post carrageenan injection in the indomethacin-treated rats same way as the test agent gemcabene.

Carrageenan-induced thermal hyperalgesia responses were measured by using a thermal nociception detection device (Hargreaves et al., 1988) described above. The glass surface temperature is maintained at 30°C. Animals were allowed to adapt to the apparatus for 10 min before initiating the protocol. The time from the onset of the heat application to paw lifting was considered to be the PWL. The examination was performed to the nearest 0.1 s of PWL. To prevent tissue damage, the cut-off latency was set at 22.5 s. Two readings were taken for each animal with 5 min spacing between readings to ensure that no summation of tissue temperature is produced. Each data point represents the mean of the 2 readings.

Gemcabene [10, 30, or 100 mg/kg in 0.5% hydroxypropyl methylcellulose (HPMC)/0.2% Tween 80] prepared in-house or vehicle (0.5% HPMC/0.2% Tween 80) were administered by oral gavage 2.5 h post carrageenan and the PWL determined 1 h post drug treatment. Drug effects are expressed as percent inhibition of the window of response between the saline treated baseline group and the carrageenan treated groups. Data were expressed as the PWL mean ± SEM.

### Monoclonal Antibody-Induced Arthritis (“MAIA”) in Female BALB/c Mice

**FIGURE 2:**
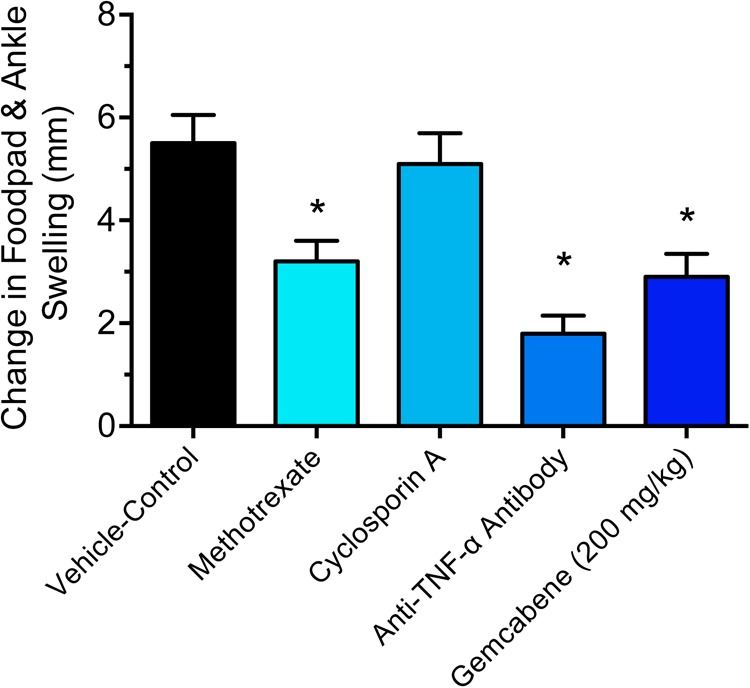
Efficacy of reference agents in the collagen antibody-induced joint swelling and arthritis mouse model. Methotrexate (4 mg/kg), cyclosporine A, and anti-TNF-α antibody (83.33 μg) were evaluated in this study. BALB/c mice (*n* = 5) were administered by oral gavage (methotrexate and cyclosporine) or intraperitoneal injection (anti-TNF-α antibody). The details are provided in the “Materials and Methods” section. The results of day 9 treatment are only shown. Data presented show changes in the footpad and ankle swelling and are expressed as mean ± SEM. ^∗^Significantly different compared to vehicle control.

We chose collagen monoclonal antibody-induced arthritis (MAIA) model as this model is a good preclinical disease model because agents that work to alleviate the human disease also work in this animal model. Since methotrexate, cyclosporin A, and anti-TNF-α antibodies have all been used in the treatment of rheumatoid arthritis in humans (Yocum et al., 1988; Weinblatt et al., 1998; Neurath et al., 1999; Weinblatt et al., 2003), so we first tested these agents in BALB/c mice using the monoclonal antibody-induced arthritis model. Methotrexate (4 mg/kg) and anti-TNF-α antibody (83.33 μg) was administered following the published methods (Williams et al., 1992; Bendele et al., 1999). BALB/c mice (*n* = 5) were administered methotrexate by oral gavage 1 h prior to the LPS injection and once daily thereafter for 9 days while anti-TNF-α antibody was administered i.p. one and 5 days after injection of Arthrogen-CIA antibody. Cyclosporin A (25 mg/kg) dissolved in olive oil was administered orally by gavage treatment for 9 days as described (Kaibara et al., 1983). The results shown in **Figure 2** demonstrated that methotrexate and anti-TNF-α antibody both significantly inhibited footpad and ankle swelling throughout the course of the disease (day 9), while cyclosporine A showed an early inhibition of the swelling response (not shown), but by day 9 there was no difference from the vehicle control. Having confirmed responsiveness of the collagen monoclonal antibody-induced joint swelling model by established agents, we used this collagen monoclonal antibody-induced arthritis (MAIA) model of joint swelling and pain in BALB/c mice as described (Sasai et al., 1999; Yumoto et al., 2002). Groups (*n* = 5) of BALB/c female mice, 6–8 weeks of age were injected with 0.4 mL of a 10 mg/mL solution of Arthrogen-CIA Monoclonal Antibody Blend (Chemicon International, Inc., Temecula, CA) intraperitoneally (IP). Two days later, the mice were injected IP with 0.2 mL of a 0.25 mg/mL solution of lipopolysaccharide (LPS from *E. coli* Strain 0111B4, St. Louis, MO, United States) in phosphate buffered Saline (PBS). Footpad and ankle size was assessed using a Dyer Digital Caliper on the day of mAb injection (Day 0), on the day of LPS injection (Day 2), and on days 4, 7, 9, and 11. Severity of the macroscopic levels of arthritis was graded after mAb injection in each of the four limbs per mouse on a 1–4 scale as described previously (Sasai et al., 1999). The criteria for the grading were as follows: 0, normal; 1, swelling and/or redness in one joint; 2, swelling and/or redness in more than one joint; 3, swelling and/or redness in the entire paw; and 4, maximal swelling. Following the development of joint swelling and arthritis, mice were administered gemcabene by oral gavage at 30, 100, or 200 mg/kg in 0.5% HPMC/0.2% Tween 80 or vehicle (0.5% HPMC/0.2% Tween 80) 1 h prior to LPS injection, and then once daily in the morning thereafter for the duration of the study.

### Monoiodoacetate (MIA) Induction of Osteoarthritis in Male Wistar Rats

Male Wistar rats (175–200 g) were housed in solid bottom isolator cages, 2–4 rats per cage, with corncob bedding on a 12:12 light:dark cycle. Animals were fed standard rat chow with water available *ad libitum*.

Osteoarthritis is a degenerative disorder of the joints that causes pain and functional disability. OA involves the articular cartilage as well as the subchondral bone, ligaments, synovial membrane, and periarticular muscle (Henrotin and Kurz, 2007; Krasnokutsky et al., 2008). The cause of OA possibly occurs from joint degeneration resulting from the combination of mechanical stress and biochemical factors. To induce OA, the animals were randomized and assigned to control and the treatment groups before the start of the study. Wistar rats were anesthetized with isoflurane and given a single intra-articular injection of MIA (1 mg) (Sigma, St. Louis, MO, United States) in physiologic saline (50 μL) through the infrapatellar ligament of the right knee using a 26.5 G needle. The contralateral control knee was injected with 50 μL of physiologic saline (Combe et al., 2004; Marker and Pomonis, 2012).

Gemcabene (**Figure 1**) was dissolved in a 0.05% hydroxypropyl methylcellulose (HPMC)/0.2% Tween 80 vehicle. Two dosing paradigms were used to test the effect of gemcabene in the Rat MIA Model: (1) A single (acute) dose was utilized to determine the effects of gemcabene, when administered acutely, on joint pain, and (2) Chronic (multiple; twice daily) dosing was utilized to determine the effect of gemcabene on cartilage structure. For the acute (single) dose paradigm, changes in hind paw weight distribution were determined on Day 14 post-MIA injection, as described previously, to establish a baseline pain reading. Rats were administered a single vehicle or a single 10, 30, or 100 mg/kg dose of gemcabene via oral gavage. Changes in hind paw weight distribution were determined 2, 4, and 6 h post compound administration. For the multiple dosing paradigm, MIA and physiological saline were injected on Day 0. Vehicle or gemcabene were administered by oral gavage, 0.5 h before MIA injection. Vehicle or gemcabene (3, 10, or 30 mg/kg) were then given approximately every 12 h for 28 days. Changes in hind paw weight distribution were determined on Days 7, 14, and 28 days. Shifts in hind paw weight distribution from the right (arthritic) to the left (contralateral control) paws were utilized as an index of joint pain and as a measure of compound efficacy (Guzman et al., 2003). An incapacitance tester (Linton Instrumentation, United Kingdom) was employed for determination of hind paw weight distribution. Each data point is the mean of 3, 5-s readings.

### Medial Tibial Plateau Erosion Analysis of Gemcabene Treated Wistar Rats With OA

After administering gemcabene b.i.d for 4 weeks, the rats were euthanized by CO_2_. The soft tissue was removed from the right (arthritic) leg, the joint was disarticulated, and the meniscus was carefully removed to expose the surface of the tibial plateau. The tibia was removed and placed in physiologic saline until erosion analysis was performed. Tibial plateau erosions were analyzed the same day as the sacrifice as follows. Each plateau was dipped in India ink for approximately 30 s to help define the erosions, rinsed in saline, and blotted on a paper towel. A Nikon stereomicroscope equipped with a digital camera was used to photograph each plateau. Two examiners graded the tibial plateaus using the following system as they were photographed: Grade 0 = No erosion; Grade I = Erosion extending into the superficial or middle layers; Grade II = Deep layer erosion; no cartilage, subchondral bone expose. The photographs were transferred to a computer for image analysis using the Zeiss KS 300 Image Analysis System (Kont Eching, Germany) to determine the total erosion area for each grade in millimeters squared. The RIDIT (Donaldson, 1998) analysis was used to determine differences in overall erosion severity. Ridit analysis is a technique to compare groups of observations and to measure the association between ordinal and nominal variables by mean ridits. By their definition mean ridits are closely related to distribution-free methods. Mean ridits are also used as a tool to test the main and/or interaction effects of factors on an ordinal response variable (Donaldson, 1998). This parameter takes into account both the erosion grade and area (small, medium, and large, quantified by dividing the area of the largest erosion in each score into thirds) simultaneously (Koh et al., 2010; Xu et al., 2012). The analysis recognizes that each unit of severity is different, but does not assume a mathematical relationship between units.

### IL-6/IL-6sR Induced Arthritis in Wistar Rats

Wistar rats were used for the IL-6/IL-6sR-induced arthritis model to evaluate efficacy of gemcabene. Wistar rats were randomized and grouped as control and treatment groups. Human recombinant IL-6 (100 ng per rat) was incubated at room temperature with human recombinant IL-6 soluble receptor (300 ng per rat) in sterile phosphate-buffered saline for 15 min prior to injection. Rats were anesthetized with isoflurane and given a single intra-articular injection (50 μL) of IL-6/IL-6sR through the infrapatellar ligament of the right knee. The contralateral control knee was injected with 50 μL sterile phosphate-buffered saline. Gemcabene was dissolved in 0.05% HPMC/0.2% Tween 80 as described above. Baseline changes in hind paw weight distribution were determined 1 day prior to injection of IL-6/IL-6sR. On the day of the experiment (day 0), a single dose of vehicle or gemcabene (10, 30, or 100 mg/kg) was administered p.o., 3 h prior to injection of IL-6/IL-6sR (*n* = 10 rats per group). Changes in hind paw weight distribution were determined 1, 3, and 6 h after injection of IL-6/IL-6sR.

### Statistical Analysis

Statistically significant differences were determined by one-way ANOVA with the Dunnett’s multiple comparisons procedure and 95% confidence interval. Data are expressed as mean ± SEM. Adequate number of animals were used for meaningful statistical significance.

## Results

### Gemcabene Reduces Carrageenan-Induced Thermal Hyperalgesia in Sprague Dawley Rat

**FIGURE 3:**
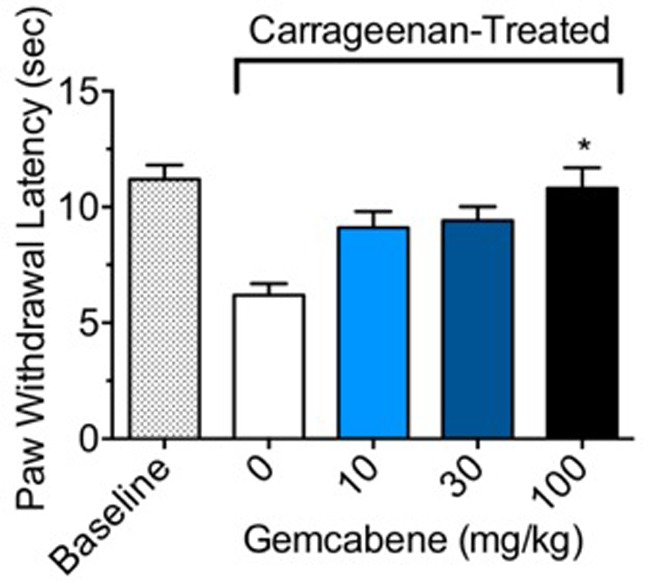
Effect of gemcabene on carrageenan-induced thermal hyperalgesia in the male Sprague Dawley rats. To induce inflammation, male Sprague Dawley rats were anesthetized and given an intraplantar injection of carrageenan (2 mg) in saline (0.1 mL). Rats (8/group) were injected with either saline (baseline) or carrageenan (control and drug treated) into the hind paw. For measurement of PWL responses, tests were done 1-h post drug administration (3.5 h post saline/carrageenan injection). Dose-related effects of gemcabene on paw withdrawal latency (PWL) was determined following exposure to radiant heat. ^∗^Significantly different from Carrageenan control. *p* < 0.05 as determined by one-way ANOVA/Tukey’s. Data are expressed as Mean ± SEM.

In carrageenan-induced inflammation, paw withdrawal latency (PWL) from a thermal stimulus can be used to measure hyperalgesia in conscious animals. In control rats without gemcabene treatment, a significant difference in PWL was noted between the non-inflamed baseline and the inflamed carrageenan-treated rats (**Figure 3**). Gemcabene administration was found to dose-dependently increase PWL time compared to the inflamed carrageenan-treated vehicle control, reaching significance at the 100 mg/kg dose (**Figure 3**). The reference agent, methotrexate showed 50% improvement in paw withdrawal latency (not shown).

### Gemcabene Attenuates Collagen Antibody-Induced Joint Swelling and Arthritis in BALB/c Mouse

**FIGURE 4:**
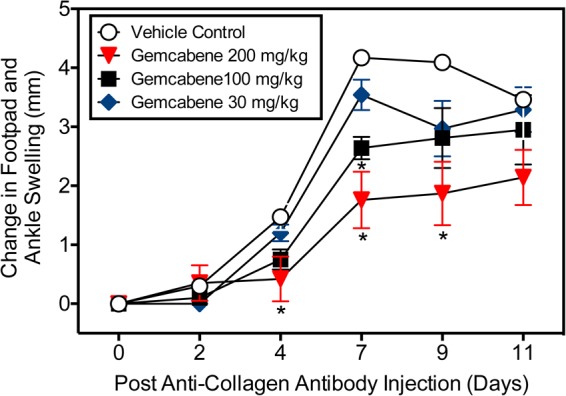
Collagen antibody-induced joint swelling and arthritis in female BALB/c mice. Joint swelling and arthritis were induced in BALB/c mice by collagen antibody injection. Mice were dosed orally with vehicle (0.5% hydroxypropyl methylcellulose/0.25% Tween-80), or gemcabene at 30, 100, or 200 mg/kg in vehicle, 1 h prior to LPS injection, and then q.d. thereafter for the duration of the study. Change in footpad and ankle swelling was measured as described in the “Experimental Procedure” section. ^∗^Indicates statistical significance (*p* < 0.05). Data are expressed as Mean ± SEM.

Intraperitoneal administration of a cocktail of mABs to type II collagen in mice induces an inflammatory arthritis condition and is used as a model of RA. A number of reference agents were evaluated in this model before carrying out studies with gemcabene (**Figure 2**). Both methotrexate and anti-TNF-alpha antibody showed expected efficacy, while cyclosporine efficacy was lost on day 9. In this model, gemcabene reduced joint swelling in the footpad and ankle in a dose- and time-dependent manner (**Table 1** and **Figure 4**). At the highest dose (200 mg/kg), gemcabene significantly inhibited footpad and ankle swelling on Day 4 (71.43% inhibition), Day 7 (57.79% inhibition), and Day 9 (54.28% inhibition) after the arthritogenic mAB injection (**Table 1**). At the intermediate dose (100 mg/kg), significant inhibition of the response occurred on Day 7 only (36.69% inhibition). At the low dose (30 mg/kg), the reduction in swelling was lower and failed to reach statistical significance. The macroscopic observations shown in **Table 2** suggest that joint swelling and paw edema improved in a dose-dependent manner corroborating the results shown in **Figure 4**. Since the time-dependent efficacy was shown up to day 7 treatment, the results of macroscopic observation for day 7 treatment are only shown (**Table 2**).

**Table 1:** Effect of gemcabene on joint swelling.

Gemcabene dose	Change in combined footpad and ankle size from day 0			
	
	Day 4	Day 7	Day 9	Day 11
	*D* (mm)	*D* (mm)	*D* (mm)	*D* (mm)
Vehicle Control	1.47 ± 0.22^a^	4.17 ± 0.32	4.09 ± 0.29	3.46 ± 0.67
30 mg/kg	1.20 ± 0.14	3.54 ± 0.26	2.97 ± 0.47	3.29 ± 0.38
100 mg/kg	0.75 ± 0.17	2.64 ± 0.19*	2.81 ± 0.51	2.95 ± 0.59
200 mg/kg	0.42 ± 0.38*	1.76 ± 0.48*	1.87 ± 0.54*	2.14 ± 0.47


*^∗^Speficies statistical significance (*p* < 0.05). ^*a*^Specifies Data are mean ± SEM.*

**Table 2:** ^a^Effect of gemcabene on severity of arthritis on day 7.

Gemcabene dose	Severity at the macroscopic level (grading 1–4)^∗^				
	
	Normal	Swelling and/or redness in 1 joint	Swelling and/or redness in >1 joint	Swelling and/or redness in entire paw	Maximal swelling
Score	**0**	**1**	**2**	**3**	**4**
Vehicle Control					**+++++**
30 mg/kg			**++**	**+++**	
100 mg/kg		**+**	**++**	**++**	
200 mg/kg		**++**	**+++**		


*^∗^1 being least severe and 4 being most severe. ^*a*^The data for each mouse (n = 5) are presented.*

### Gemcabene Attenuates Changes in Hind Paw Weight Distribution in the Rat MIA Model of Osteoarthritis

#### Dose Selection in Chronic Study

**FIGURE 5:**
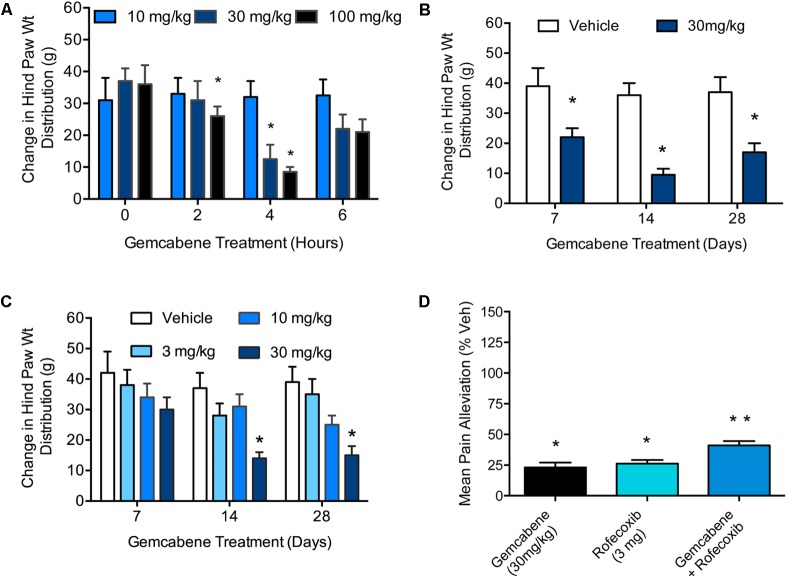
Effect of gemcabene on monosodium iodoacetate (MIA)-induced change in hind paw weight distribution. **(A)** MIA was injected into the right knee and saline into the left knee of all rats on Day 0. On day 14 the rats were assessed on an incapacitance tester and then given gemcabene (10, 30, or 100 mg/kg, po). Two, 4, and 6 h later, the rats were reassessed. Statistically significant differences were determined by one-way ANOVA followed by Dunnett’s multiple comparisons procedure. Data are expressed as mean ± SEM, *N* = 8 rats per group. **(B)** MIA and saline were injected on day 0. Gemcabene (30 mg/kg) was given p.o., 0.5 h before MIA injection. Gemcabene was then given b.i.d for 28 days. Changes in hind paw weight distribution were determined on Days 7, 14, and 28. Statistically significant differences were determined by one-way ANOVA with the Dunnett’s multiple comparisons procedure (^∗^*p* < 0.05). Data are expressed as mean ± SEM. *N* = 12 rats per group. **(C)** 28 days dose-response study. MIA and saline were injected on day 0. Gemcabene (3, 10, or 30 mg/kg) was given PO, 0.5 h before MIA injection. gemcabene was then given b.i.d for 28 days. Changes in hind paw weight distribution were determined on days 7, 14, and 28. **(D)** Rats were treated with MIA as shown in **A** for 14 days followed by acute treatment with the reference agents. Only 4 h time points are shown. Data presented show pain alleviation compared to vehicle control (100% pain, 0% comfort). Statistically significant differences were determined by one-way ANOVA with the Dunnett’s multiple comparisons procedure (^∗^*p* < 0.05). Data are expressed as mean ± SEM. *N* = 12 rats per group. ^∗∗^*p* < 0.01.

A MIA model of OA induction in rats was used whereby hind paw weight distribution was measured to evaluate gemcabene efficacy. First, MIA was injected into the right knee and saline into the left knee of all rats on Day 0. On day 14, the rats were assessed on an incapacitance tester followed by oral administration of 10, 30, or 100 mg/kg of gemcabene. After 2, 4, and 6 h, the rats were reassessed for paw weight distribution. The two higher gemcabene doses (30 and 100 mg/kg) showed a peak response at 4h that waned by 6 h (**Figure 5A**). Since 30 and 100 mg/kg doses showed efficacy in this acute single dose experiment, we carried out a chronic repeat-dose study up to 28 days. In this experiment (day 0), gemcabene (30 mg/kg) was orally administered 0.5 h before MIA and saline injections in the right and left knees, respectively. Thereafter, gemcabene was administered b.i.d. by oral gavage for 28 days at 30 mg/kg/day. Changes in joint discomfort were measured by changes in hind paw weight distribution. As shown in **Figure 5B**, gemcabene significantly decreased the change in hind paw weight distribution at all three time-points tested (47, 71, and 60%, inhibition at days 7, 14, and 28, respectively; *p* < 0.05; Figure 3). A dose response for gemcabene was also performed to look at joint discomfort as measured by changes in hind paw weight distribution after multiple dosing (**Figure 5C**). Treatments with gemcabene at 30 mg/kg significantly decreased the change in hind paw weight distribution on days 14 and 28 (50 and 62% inhibition, respectively; *p* < 0.05; **Figure 5C**). However, the significant effect observed acutely in the first experiment at 30 mg/kg was not observed on day 7. There were no significant changes in hind paw weight distribution at the 3 and 10 mg/kg doses at all three time-points (7, 14, and 28 days) tested (**Figure 5C**). We compared rofecoxib, a COX-2 inhibitor, with gemcabene either alone or in combination and the results shown in **Figure 5D** suggest that 3 mg/kg dose of rofecoxib was comparable to 30 mg/kg gemcabene in acute hyperalgesia model. Combination of rofecoxib and gemcabene showed additive efficacy.

### Gemcabene Attenuates Medial Tibial Plateau Erosion Severity and Area in the Rat MIA Model of Osteoarthritis

#### Experiment 1: A Single Concentration of Gemcabene (30 mg/kg)

Gemcabene was tested for the ability to preserve cartilage structure in the rat MIA model described above. Rats were orally dosed 30 mg/kg, b.i.d. for 28 days and sacrificed on day 28. The tibial plateaus were removed and analyzed as described in the Section “Materials and Methods.”

**FIGURE 6:**
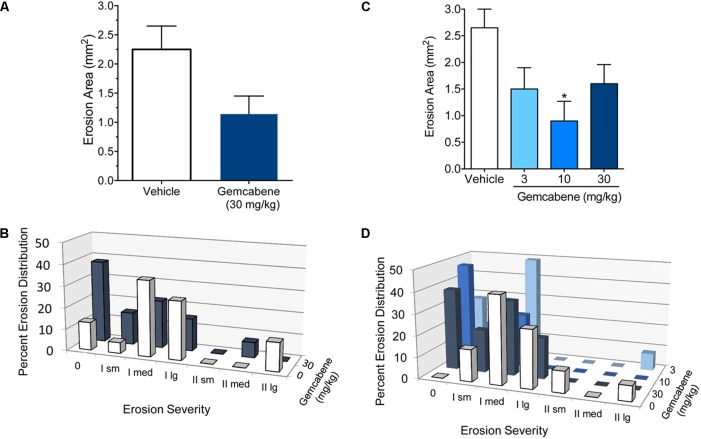
Effect of emcabene on medial tibial plateau erosion area in the rat MIA model of OA. **(A)** MIA and saline were injected on day 0. Gemcabene (30 mg/kg) was given p.o., 0.5 h before MIA injection and was then given b.i.d for 28 days. Erosion analysis was performed on day 28. There were no statistically significant differences as determined by *t*-test (*p* = 0.179). *N* = 12 rats per group. **(B)** Effect of Gemcabene (3, 10, and 30 mg/kg) on medial tibial plateau erosion area in the rat MIA model of OA. MIA and saline were injected on day 0. Gemcabene (3, 10, or 30 mg/kg) was given p.o., 0.5 h before MIA injection and was then given b.i.d for 28 days. Erosion analysis was performed on day 28. Only the 10 mg/kg group was statistically significant as determined by one-way ANOVA, Dunnett’s multiple comparisons procedure (*p* < 0.05). *N* = 12 rats per group. **(C)** Percent erosion distribution from experiment in **(A)** with 30 mg/kg/d dose. **(D)** Percent erosion distribution from experiment in **(B)** with dose-response (10, 30, and 100 mg/kg). Data are expressed as Mean ± SEM.

The effect of gemcabene on total erosion area (regardless of grade) decreased the total erosion area by 43%, however, this decrease was not significant and may in part be due to two rats in the vehicle group showing no erosions despite MIA treatment (**Figure 6A**). In this same experiment, gemcabene had no significant effect on tibial plateau erosion severity when analyzed using the RIDIT test (*p* = 0.10; **Figure 6B**). However, it should also be noted that 5 of the 12 rats treated with gemcabene had no erosions.

#### Experiment 2: Gemcabene Dose-Response (3, 10, and 30 mg/kg)

In a second experiment, rats were subjected to MIA treatment as in the experiment above and were orally dosed with 3, 10, or 30 mg/kg gemcabene, b.i.d. for 28 days and sacrificed on day 28. The tibial plateaus were removed and analyzed for total erosion area regardless of grade. Although all doses showed reduction in total erosion area, a significant decrease was noted only at the 10 mg/kg gemcabene dose (62.4%; *p* < 0.05) (**Figure 6C**). Tibial plateau erosion severity was determined using the RIDIT test. All three doses of gemcabene showed effect on erosion severity (**Figure 6D**).

### Gemcabene Improves Hind Paw Weight Distribution in the Rat IL-6/IL-6sR Knee Injection Model

IL-6 and IL-6 soluble receptors are associated with the severity of rheumatoid arthritis and cause severe destruction of cartilage and bone (Robak et al., 1998; Sanchez et al., 2004). Interleukin-6 (IL-6) alone does not induce osteoclast formation, but soluble interleukin-6 receptors (sIL-6R) triggers the formation in the presence of IL-6 (Kotake et al., 1996). Therefore, we used IL-6 plus sIL-6R administration in the right knee to induce arthritis in rats. Phosphate-buffered saline was injected in the left knee to serve as control. The change in hind paw weight distribution was used as a measure of joint discomfort and efficacy of gemcabene. We carried out two separate studies, one with three doses of gemcabene (10, 30, and 100 mg/kg/d) given one time and measurements made up to 6 h. The other study was carried out with two doses of 10 and 30 mg/kg/d gemcabene.

**FIGURE 7:**
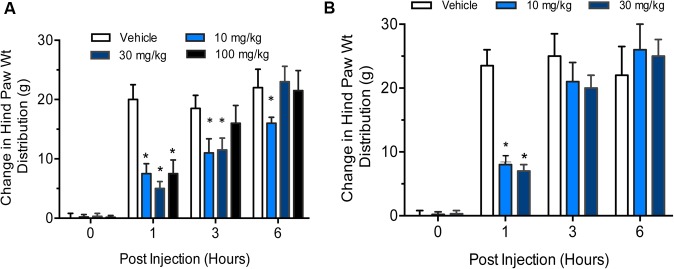
Effect of gemcabene on change in hind paw weight distribution in the rat IL-6/IL-6sR knee injection model. **(A)** Experiment 1 Baseline incapacitance readings were taken on Day –1. IL-6/IL-6sR and PBS were injected on day 0. Gemcabene (10, 30, or 100 mg/kg) was given p.o. 3 h before IL-6/IL-6sR injection. Changes in hind paw weight distribution were determined 1, 3, and 6 h post-injection (4, 6, and 9 h post-gemcabene dose). Statistically significant differences were determined by one-way ANOVA followed by the Hochberg’s procedure (^∗^*p* < 0.001). Data are expressed as mean ± SEM. *N* = 10 rats per group. **(B)** Experiment 2 was carried out same way as experiment 1, but at only two doses, 10 and 30 mg/kg. The results show reproducibility of the findings.

In the first experiment, all doses of gemcabene significantly decreased the change in hind paw weight distribution at 1 h post-injection, 4 h post- gemcabene administration (53 ± 9%, 74 + 8%, and 57 + 15% inhibition at 10, 30, and 100 mg/kg, respectively; *p* < 0.001; **Figure 7A**). No significant effect was noted at 3 h post injection in 100 mg dose group and 6 h post injection in 30 and 100 mg dose groups (6 and 9 h post- gemcabene administration).

In the second experiment, gemcabene was tested only at 10- and 30 mg/kg and all incapacitance test readings were blinded. Both the 10- and the 30-mg/kg doses of gemcabene significantly decreased change in hind paw weight distribution 1 h post-injection as compared to the vehicle group (63 + 6% and 71 + 10% inhibition at 10 and 30 mg/kg, respectively; *p* < 0.001; **Figure 7B**).

## Discussion

To investigate the anti-inflammatory properties of gemcabene, we assessed gemcabene’s anti-inflammatory efficacy in multiple rat and mouse models of pain and OA (van der Kraan et al., 1989; Marcelletti et al., 1991; Guingamp et al., 1997; Guay et al., 2004; Teeple et al., 2013). Gemcabene was found effective in preventing thermal hyperalgesia induced by carrageenan footpad inflammation (Guay et al., 2004). At the 100 mg/kg dose, gemcabene significantly increased the PWL as compared to vehicle-treated animals (**Figure 3**), indicating its anti-nociceptive or analgesic properties in the setting of inflammatory pain (Hargreaves et al., 1988). These results demonstrate that gemcabene is an orally effective anti-nociceptive agent and may therefore represent a potentially useful pharmacological approach for the treatment of diseases characterized by increased nociception. Conventional NSAIDs, COX-2 inhibitors and prostaglandin E2 (PGE2) monoclonal antibodies are effective anti-inflammatory agents in these models (Chan et al., 1995; Zhang et al., 1997). Indomethacin is also known to block COX-2 induction in paw edema, but not in hyperalgesia suggesting that a positive feedback loop regulates COX-2 expression in the paw edema model (Nantel et al., 1999).

To further evaluate the efficacy of gemcabene’s anti-inflammatory activity, we assessed its activity in the mouse model of collagen antibody induced arthritis (Khachigian, 2006; Yasuda, 2006; Nasu et al., 2008). This model is an effective approach to interrogating effector cell signaling and function in an arthritic animal. The mAb-induced arthritis model responds well to most anti-arthritic compounds that work in CIA. Gemcabene was significantly efficacious in the mAb-induced arthritis model at 100 and 200 mg/kg doses, but not at 30 mg/kg dose (**Figure 4**) This compares well to gemcabene activity in rodent models of lipid regulation, where 30 and 100 mg/kg gemcabene was required to see a pharmacological effect (Bisgaier et al., 1998). The ability of gemcabene to decrease pain related behaviors in models of pain and arthritis suggest that the compound may have clinically relevant effects on pain conditions. In the paw edema model, COX-2 levels are elevated with a concomitant increase in prostaglandin production (Seibert et al., 1994; Zhang et al., 1997). Cyclooxygenase-2 (COX-2) levels are elevated in conditions of paw edema and attenuation of hyperalgesia have been reported by COX-2 inhibitors (Chan et al., 1995; Zhang et al., 1997). This suggests that COX-2 may be one of the targets for gemcabene-mediated efficacy. To further gain insights into the role of COX-2 in gemcabene-mediated attenuation of hyperalgesia, we carried out studies with COX-2 inhibitor, rofecoxib and gemcabene either individually or in combination and the results shown in **Figure 5D** suggest that most likely the efficacy of gemcabene occurs via mechanism other than inhibition of COX-2, since additive efficacy was observed when animals were treated with the combination of gemcabene and rofecoxib. Although gemcabene modulates some of the PPAR target genes in animal model (Bisgaier et al., 1998), a thorough investigation using transactivation assays did not show appreciable PPAR agonist or antagonist activities (Bisgaier et al., 2018). PPAR-α and PPAR-δ agonists are known to have anti-inflammatory activities (Gonzalez et al., 2009; Bojic et al., 2014), but gemcabene does not appear to have anti-inflammatory activities through PPAR activation. Cell-based studies with gemcabene indicated that gemcabene down-regulates CRP gene expression through down-regulation of C/EBP-δ and NF-κB promoter activities (Srivastava et al., 2018). Anti-inflammatory activities of many molecules are attributed to down-regulation of NF-κB (Srivastava et al., 2015). Thus, gemcabene-mediated down-regulation of NF-κB, in part, appears to have contributed to its anti-inflammatory activity.

In the MIA induced rat model of OA (Guingamp et al., 1997; Guzman et al., 2003; Combe et al., 2004), a single dose of gemcabene at 30 mg/kg effectively improved joint pain in arthritic rats, suggesting that the effects of this compound may be related to a direct antihyperalgesic effect. Gemcabene also improves joint pain in the same rat MIA model in a dose-dependent manner when given chronically (**Figure 5B**). Gemcabene administered at 30 and 100 mg/kg improved joint pain after 1–2 weeks of dosing with sustained effects up to 4 weeks. In addition, this compound effectively decreased medial tibial plateau erosion size when administered at 3, 10, or 30 mg/kg, for 28 days (**Figure 6**). In RA rat model using IL-6/IL-6sR knee injection, gemcabene significantly attenuated joint pain 1 h post-injection indicating a potential mechanism of action for gemcabene (**Figure 7**). The levels of IL-6 and its receptor are elevated in synovial fluid and serum in humans with rheumatoid arthritis (Kotake et al., 1996; Robak et al., 1998). Thus, in a number of animal models of inflammatory diseases, gemcabene showed efficacy, which corroborate cell-based findings on inflammation-induced inhibition of CRP production by gemcabene (Srivastava et al., 2018). The attenuation of disease severity in animal models of pain in the present study further demonstrates gemcabene as an anti-inflammatory agent.

Based on our present results, we demonstrate that gemcabene is an anti-inflammatory agent and attenuates inflammatory disease in animal models. These findings are consistent with the results in clinical studies showing reduction of pro-inflammatory protein CRP (Stein et al., 2016) and in cell models showing inflammation-induced reduction of CRP mRNA and protein (Srivastava et al., 2018). Attenuation of disease severity by gemcabene in multiple inflammatory disease models reported here further support gemcabene as a potential anti-inflammatory agent. Given the inflammatory nature of atherosclerotic plaque formation, progression and destabilization (Cybulsky et al., 2001; Libby, 2002; Stoll and Bendszus, 2006), it is anticipated that gemcabene may prove to be a better agent in cardiovascular disease (CVD) attenuation compared to agents with only antihyperlipidemic properties (Srivastava et al., 2006; Armitage et al., 2010; Ballantyne et al., 2013; Pinkosky et al., 2013; Song and FitzGerald, 2013). Indeed, a recent clinical trial, CANTOS (Canakinumab Anti-inflammatory Thrombosis Outcomes Study) (Ridker et al., 2017), with an anti-inflammatory agent demonstrated the benefits of anti-inflammatory agents in attenuating cardiovascular events. Inhibition of IL-1β-induced inflammation (Ridker et al., 2017), CRP production by gemcabene (Srivastava et al., 2018), and *in vivo* efficacy in animal models of inflammation in the present study suggest that anti-inflammatory agents may improve CVD events. Thus, gemcabene’s anti-inflammatory property together with LDL lowering offers added benefit to CVD patients.

## Author Contributions

RS evaluated data from the studies and has contributed to the interpretation and analyses of data as well as writing of the manuscript. JC and BM supervised, conducted, and evaluated the research in the studies and have contributed to the review of the manuscript. CB evaluated data from the studies, has contributed to the writing of the manuscript, and is the co-inventor of Gemcabene.

## Conflict of Interest Statement

RS is employed and has ownership interest in Gemphire Therapeutics Inc. JC is currently employed at Charles River Laboratories International, Wilmington, MA and BM at Diapin Therapeutics, Ann Arbor, MI. Both these authors have no conflicts of interest. CB is employed at Gemphire Therapeutics and has ownership interest in Gemphire Therapeutics Inc.

## Abbreviations

CAIA: collagen antibody-induced arthritis
CIA: collagen-induced arthritis
CITH: carrageenan-induced thermal hyperalgesia
JUPITER: justification for the use of statins in primary prevention: an intervention trial evaluating rosuvastatin
MIA: monosodium iodoacetate
OA: osteoarthritis
PWL: paw withdrawal latencies
